# Evaluating the impact of large-scale nucleic acid testing and home quarantine on a novel emerging infectious disease prevention and control: a dynamic modeling approach

**DOI:** 10.3389/fpubh.2025.1447738

**Published:** 2025-05-19

**Authors:** Zuiyuan Guo, Yuheng Chen, Guangquan Xiao, Huawei Jiang, Zurong Yang, Jiangfan Li, Lili Gong, Meisong Jin, Feng Wang

**Affiliations:** ^1^The First Department of Infectious Disease Prevention and Control, Center for Disease Control and Prevention in Northern Theater Command, Shenyang, China; ^2^College of Communication Engineering, Jilin University, Changchun, China; ^3^Unit 30, PLA 93286 Troops, Shenyang, China; ^4^Department of Disease Surveillance, Center for Disease Control and Prevention in Northern Theater Command, Shenyang, China; ^5^Department of Epidemiology, School of Public Health, Air Force Medical University, Xi’an, China; ^6^Department of Pediatrics, General Hospital of Northern Theater Command, Shenyang, China; ^7^Department of Health Supervision and Surveillance, Center for Disease Control and Prevention in Northern Theater Command, Shenyang, China; ^8^Department of Pest Control and Medical Infection Control, Center for Disease Control and Prevention in Northern Theater Command, Shenyang, China

**Keywords:** respiratory infectious disease, COVID-19, dynamic model, nucleic acid testing, home quarantine, basic reproduction number

## Abstract

**Introduction:**

Conducting large-scale viral nucleic acid testing and isolating SARS-CoV-2-infections were crucial strategies in China, which played a key role in successfully controlling multiple waves of the Omicron epidemic. To thoroughly analyze the mechanisms and value of these measures, including testing and isolation, in epidemic prevention and control, and to provide a theoretical basis for scientific epidemic prevention and precise strategies in the face of potential future outbreaks of novel respiratory infectious diseases.

**Methods:**

We developed an individual-based computational model of infectious disease dynamics. The model simulates regular large-scale nucleic acid testing for community residents during an epidemic. When individuals tested positive, they and their household members, as close contacts, are subjected to home isolation. During home isolation, the virus is assumed not to spread outside the household, but the potential for transmission within the household remained. Isolation measures can be lifted once the testing results turned negative. Finally, sensitivity analysis was conducted to verify the scientific validity and reliability of the model.

**Results:**

The study found that the efficacy of testing and isolation in epidemic prevention is closely related to the speed of disease transmission. When the basic reproduction number (*R0*) is less than 3, these measures can significantly reduce the infection rate among the population and the speed of epidemic spread; otherwise, they fail to achieve the goal of controlling the epidemic.

**Discussion:**

Reducing person-to-person contact is crucial for epidemic prevention and control. In addition to testing and isolation, comprehensive non-pharmaceutical interventions (NPIs) should also be implemented, such as increasing social distancing, restricting gatherings in public places, and promoting vaccination, to control the transmission of the epidemic.

## Introduction

Respiratory infectious diseases can emerge suddenly, exhibiting strong infectivity and producing a large number of infections within a short time span. This leads to a surge in severe cases and deaths, overburdening medical resources. During the COVID-19 pandemic, Mainland China employed strict measures for the nucleic acid testing of SARS-CoV-2 infected individuals at early stages, as well as for reporting, isolation, and treatment (1). During the 2022 Omicron variant outbreak, actively encouraging nucleic acid testing for residents in affected areas and the immediate isolation of identified cases and their close contacts—key strategies collectively referred to as “testing and isolation”—are essential measures for effective epidemic prevention and control. These strategies, in conjunction with other measures, successfully mitigated the impact of multiple waves of the epidemic.

RT-PCR, a rapid and accurate method for virus nucleic acid testing, has played a significant role in epidemic prevention and control (2–5). During the Omicron pandemic, the Chinese government regularly assessed the infection status of its population by encouraging residents to voluntarily undergo testing. After laboratory testing confirmed infections, local Centers for Disease Control and Prevention (CDC) would directly report relevant information through the disease control and prevention information system within specified time intervals (1). Simultaneously, asymptomatic infected individuals and cases would be transported to designated medical institutions or government-established shelter hospitals for isolation and treatment to prevent epidemic spread. Epidemiological investigations would then be conducted to identify close contacts. Due to their high risk of infection, a combination of centralized and home quarantine observation was adopted for these close contacts (1). Infected individuals and close contacts underwent regular testing during isolation. Once the infections were confirmed negative and close contacts were excluded from infection, they would be released from isolation for treatment or observation.

The control of an epidemic necessitates the simultaneous implementation of comprehensive NPIs. Due to the enhanced transmissibility of the Omicron variant (6), the duration of these measures, such as increased social distancing and travel restrictions, has been extended, which undeniably has substantial negative impacts on economy and human interaction. The aim of this study is to search for a measure that has a relatively lower social cost, which can not only effectively suppress the virus transmission but also minimize the socioeconomic impact of NPIs. Drawing on China’s experience in controlling Omicron, we strive to further investigate the effectiveness of testing and isolation in containing the epidemic. Moreover, by establishing a model, we aim to demonstrate whether relying solely on this measure can effectively control the epidemic. Thus, it is essential to examine the relationship between the speed of epidemic transmission (typically measured by *R0*) and testing and isolation strategies.

Although numerous publications have explored mathematical models for analyzing NPIs, such as traffic restrictions, isolation measures, and immunization (7–12), there is a scarcity of studies addressing large-scale testing and isolation in populations. Among them, Zhu WL developed a branching process model and a differential equation-based compartment model to simulate the transmission dynamics of COVID-19, assessing the impact of extensive nucleic acid testing and isolation measures on the epidemic. It has been found that prompt serial nucleic acid testing may effectively contain an outbreak. Moreover, a decrease in the isolation period and effectiveness of comprehensive quarantine measures resulted in an increase in the cumulative number of cases and an extension of the epidemic duration (13, 14). Additionally, Kucharski AJ utilized a mathematical model to simulate the effect of a variety of testing, isolation, tracing, and physical distancing scenarios when *R0* is 2.6. The study found that if combined with moderate physical distancing measures, isolation and contact tracing strategies would reduce transmission to a greater extent than mass testing or self-isolation alone (15). These studies have demonstrated the impact of testing and isolation on the magnitude of an epidemic. However, it remains necessary to establish a model that is more aligned with China’s epidemic prevention policies to investigate this issue further and to explore whether this approach alone can contain the prevalence of rapidly spreading pathogen.

To address these scientific issues, we developed a computer model to simulate the spread of infectious diseases in a population after implementing testing and isolation measures, and quantitatively analyzed their impact on the epidemic trends. To verify the effectiveness of the strategy, our model not only included testing and isolation measures solely but also replaced the admission of asymptomatic and mild cases to designated hospitals or shelter hospitals with home isolation treatment. Furthermore, the definition of close contacts has been further narrowed (limited only to family members of the infected individuals), and the practice of centralized quarantine observation has been shifted to home quarantine observation.

The stochastic computer model, with individuals as the research unit, fully translates mathematical formulas into program code. It conducts computer simulations based on pathogen transmission through person-to-person contact in the real world, enabling the analysis of complex human activities and their relationships with infectious disease epidemic trends. We have employed this method multiple times to simulate the spread of respiratory infectious diseases, examining the dynamic relationships between behaviors such as population outflow, asymptomatic carrier screening, household gatherings, cross-city travel, elevator usage, and bus transportation, and the progression of the epidemic (16–21). Experience has demonstrated that, compared to traditional dynamic models that use differential equations as research methods, this approach can more effectively describe individual behaviors. This advantage allows us to gain deeper insights into the mechanisms through which complex human behavior and prevention measures affect epidemic spread.

In the present study, we used this method to establish a stochastic compartment model to simulate the epidemic transmission process among households. Drawing on China’s experience and practices in addressing the Omicron variant, we conducted an in-depth analysis of the effectiveness of testing and isolation in epidemic prevention and control. Our research outcomes can provide a theoretical foundation for future scientific responses to emerging respiratory infectious diseases.

## Materials and methods

### Data and parameters

The parameters utilized in the model were primarily derived from published literature, encompassing the incubation period, the duration of positive testing, sensitivity of testing, and the population distribution of Chinese households. Furthermore, some parameters were based on the authors’ assumptions drawn from our fieldwork experience in COVID-19 prevention and control efforts, as presented in Table 1.

**Table 1:** Model parameters.

Description	Distribution characteristics	Numerical values	Sources
Total number of homes	Constant	500	Assumed
Incubation period (days)	Lognormal distribution	*μ* = 3.1 *σ* =2.6	(30)
Duration from testing positive to negative (from the first positive to the negative nucleic acid test result, in days)	Normal distribution	*μ* = 8.4 *σ* = 4.8	(31)
Proportion of innate immune population, *q*	Constant	0.1	Assumed
Proportion of people refusing testing, *p*	Constants	0.1, 0.2, 0.3	Assumed
Period of testing, *T* (days)	Constant	1, 2, 3	Assumed
Sensitivity of testing	Constant	0.87	(32)
Specificity of testing	Constant	1.00	(33)
Extra-household adequate contact rate, *λ*	Constant	1, 2, 3	Assumed
Hospitalization rate	Constant	0.02	(34)
Duration of hospitalization (days)	Normal distribution	*μ* = 6.35 *σ* = 2.5	(31)
Distribution of Chinese household sizes	Constants	Probabilities that the household size was 1, 2, 3, and 4 were 0.2, 0.33, 0.28, and 0.19, respectively	(35)

### Model preconditions

**Figure 1:**
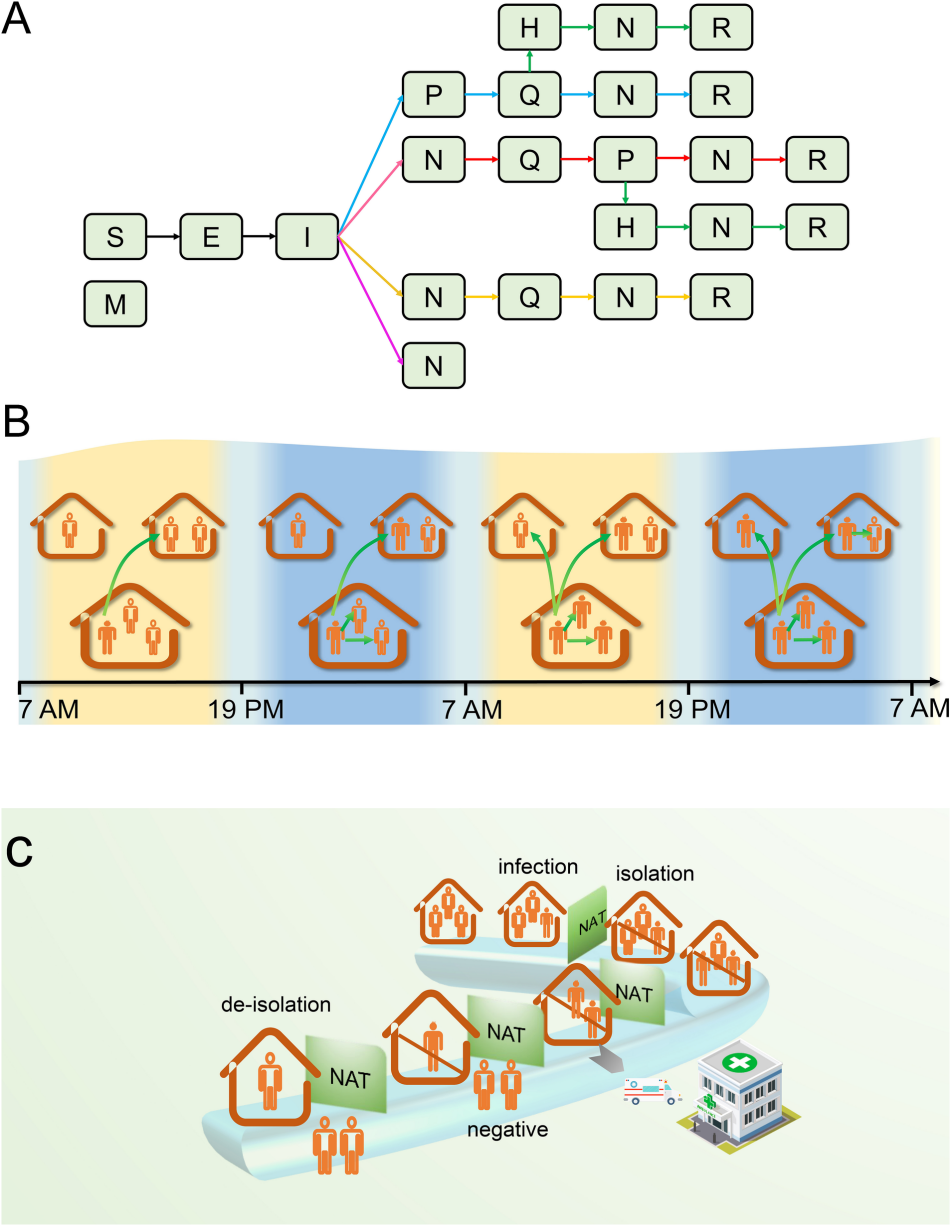
The transmission process of the epidemic within a household. **(A)** There are five possible scenarios of infection state transition that may exist for the first infector in a household. If the infector tested positive after becoming I: (1) they will be placed under home quarantine; during the quarantine period, they may be hospitalized for treatment due to worsening of their condition; after turning negative, they will be released from quarantine (green arrow); (2) during home quarantine, they are not hospitalized for treatment, and are released from quarantine after turning negative (blue arrow). If the infector’s testing is negative or they refuse testing, they may not be quarantined temporarily but might be quarantined as close contacts due to other household members testing positive. It can be manifested in the subsequent three scenarios: (3) they may test positive after isolation and are released after turning negative (red arrow) or transferred to the hospital for treatment and discharged upon recovery; (4) no positive results are detected after quarantine, and quarantine is lifted after a maximum incubation period (yellow arrow); (5) both the infector and their household members refuse to be tested or do not test positive, and the household is not quarantined (pink arrow). **(B)** Transmission between and within households. **(C)** The chronological order of infection, testing, isolation, and release of isolation in a household. S, susceptible; M, immune; E, exposed; I, infectious; P, positive; H, hospitalized; Q, quarantined; N, negative; R, recovered; NAT, nucleic acid test.

Population social activities and infectious disease prevalence are complex and uncertain. To eliminate unnecessary interference and maintain a focused, concise model, it is crucial to establish certain model assumptions. Building upon China’s practical experience, we refined the assumptions into the following points:

(1) A closed small town was selected as the epidemic outbreak location, consisting of 500 households and 1,230 residents. Due to the short epidemic period, population migration, birth, and natural death were not considered. Based on the fact that Omicron’s severity is significantly lower than that of Delta (22), the model did not take into account death cases. It was assumed that a proportion (*q*) of residents possessed innate immunity to SARS-CoV-2, while the remaining residents were susceptible.
(2) Compartments in the model could be divided into 9 categories: immunized (M, having innate immunity), susceptible (S), exposed (E, incubating and being not yet contagious), infectious (I, being contagious), positive (P, positive test result), quarantined (Q, home quarantine), hospitalized (H, being hospitalized), negative (N, test turning negative), released (R, released from quarantine). The transformation relationship between them was shown in Figure 1A.
(3) Patient zero was infected at time *t* = 0, selected at random from a cohort of 500 households. Numerous studies suggest that an infected individual may cause intra-household infections during the infectious period (23, 24), thus warranting a distinction between infections occurring inside and outside the household within our model. To evaluate the disease transmission capability of an infector, we defined parameter *λ_t_* as the extra-household adequate contact rate, representing the daily number of effective contacts between an infector and residents of other households at time *t*. This parameter reflects the residents’ mobility, social environment, and the pathogen’s transmissibility. As the outbreak evolves and increasing numbers of people are isolated, the number of effective contacts will decrease in response to the rising count of isolated individuals. We posit that on day *t*, the number of individuals an infector contacts outside the household follows a Poisson distribution, with an average *λ*(1*-r*(*t*)), where *λ* indicates the initial stage extra-household adequate contact rate, and *r*(*t*) represents the proportion of the population that is isolated compared to the total population at time *t*.
(4) Considering that most residents venture out during the day for work or school and return home in the evening, the timing for extra-household infections is designated as between 7:00 and 19:00 daily, occurring exclusively among non-isolated residents. Intra-household infections are set to take place from 19:00 until 7:00 the following day, while infections within isolated households can be initiated at any time. The association between the timing and the location of infection events is delineated in Figure 1B.
(5) To promptly identify infectors, the government organized a nucleic acid screening among town residents at intervals of *T* days. Since the sensitivity of testing is less than 1, infectors might require multiple tests to be detected, or they might not be detected until their test results turned negative. Although the majority of residents complied with the government’s recommendation for regular testing, a proportion (*p*) of residents refused testing when their households were not in quarantine for various reasons.
(6) Testing and the acquisition of test results occurred daily from 8:00 to 17:00. Upon the first confirmation of a positive household member, the CDC would enforce home isolation treatment for the infected individual by 19:00 on the same day of detection. Concurrently, as close contacts, other household members would also be subject to home quarantine observation along with the infected individual. Susceptible individuals will inevitably become infected during home quarantine, since infectors and susceptible household members share the same living space 24 h a day. The government guarantees the provision of daily essentials for quarantined homes; consequently, individuals do not need to leave their homes for shopping during the isolation period.
(7) After homes were isolated, the staff from the CDC would visit these homes every *T* days to collect nucleic acid samples from all isolated households (including infectors and close contacts) for laboratory testing starting from the second day of isolation, and also count the number of positive cases. In an effort to minimize the duration of the quarantine, once an infector’s test results turned negative, their quarantine would be lifted. However, if any other household members were still infected, the quarantine would continue until they tested negative. Throughout the home quarantine period for infectors, a limited number of critically ill patients would be immediately transferred to hospitals for isolation and treatment. Owing to the strict personal protection measures implemented by medical staff, no epidemic spillover would occur in the hospital. Hospitalized patients who tested negative could be discharged from the hospital. The chronological sequence of these events is intuitively illustrated in Figure 1C.

### Model design framework

In the model program, we first established the basic parameters, including incubation period, duration from testing positive to negative, and extra-household adequate contact rate, among others. Then, the member IDs of each household, the randomly generated IDs of immune individuals, and the IDs of individuals who refuse testing were stored in separate data frames, the code is detailed in Supplementary Appendix 1, lines 5–55. After that, we proceed to simulate a complete transmission process of the epidemic, which is mainly divided into the following four steps.

Step 1: Set up the index case. Randomly select a non-immune infected individual who voluntarily undergoes testing as the index case (Patient 0). Store five elements: the location of infection (within or outside the household), the household ID, the source of infection (not applicable here), the infected individual’s ID, and the time of infection, in a dataframe named State_t. Initially, this dataframe contains only one column (Supplementary Appendix 1, lines 57–90).

After completing the above setup, begin the epidemic simulation based on the order of infection time. First, locate the column in State_t with the earliest infection time, extract the information from this column, and then remove it. If the infection occurs within the household, the infected individual’s ID can be directly retrieved from this column. If the infection occurs outside the household, randomly select a non-isolated resident from all exposed individuals. If this resident is susceptible, they become infected, and their information (including the source of infection, their own ID, their household’s ID, the location of infection, the time of infection, the time when they become infectious, the time when their test turns negative, etc.) is stored in another dataframe for statistical analysis of time distribution after the epidemic simulation concludes (Supplementary Appendix 1, lines 92–153).

To sustain the epidemic, identify the next generation of infected individuals using the current one as the source of infection. Therefore, we must determine the infection times for subsequent generations. Since these time points are related to the isolation time of the household where the source of infection resides, the isolation time must be determined first.

Step 2: Determine the household isolation time. If the infected individual is a resident who voluntarily undergoes testing, generate a random number based on a geometric distribution with the test sensitivity as the parameter. This random number represents the total number of tests until a positive result is obtained, and it is used to estimate the time of the positive test result, providing a basis for further determining the start of the isolation period. If the first infected individual in the household voluntarily undergoes testing, two scenarios may arise based on the test results: First, if no positive result is ever detected, the household isolation time is determined by the test results of other members. Second, if a positive result is detected, the household isolation time can be tentatively determined. However, if a positive test result for another member precedes the previously determined isolation time, the household isolation time needs to be adjusted accordingly to an earlier date. Since the model calculates each infected individual sequentially, this situation is possible.

Step 3: Determine the extra-household infectious period. When the isolation time is advanced, the extra-household infectious period for each member may also be correspondingly shortened, necessitating a comparison and update for each member. Here, it is necessary to separately analyze the infected household members who refuse testing and those who voluntarily undergo testing. Finally, since the shortening of the infectious period may lead to a reduction in the number of infections, the columns corresponding to these secondary infections need to be removed from the State_t. The code for Steps 2 and 3 can be found in Supplementary Appendix 1, lines 154–288.

The above two steps analyze the scenario where infected individuals voluntarily undergo testing. If they refuse, the calculation of hypothetical positive time and infectious period is similar, see Supplementary Appendix 1, lines 289–326 for details.

Step 4: Determine infection times. After determining the extra-household infectious period, calculate the number and infection times of secondary infections both within and outside the household, and append this information to State_t (columns increase then decrease to zero, indicating epidemic end). Separate calculations are required due to differing infection time periods. Household secondary infections can be directly assigned identifiers (Supplementary Appendix 1, lines 327–544), while outside infections require random selection from non-isolated residents as described in Step 1.

**Figure 2:**
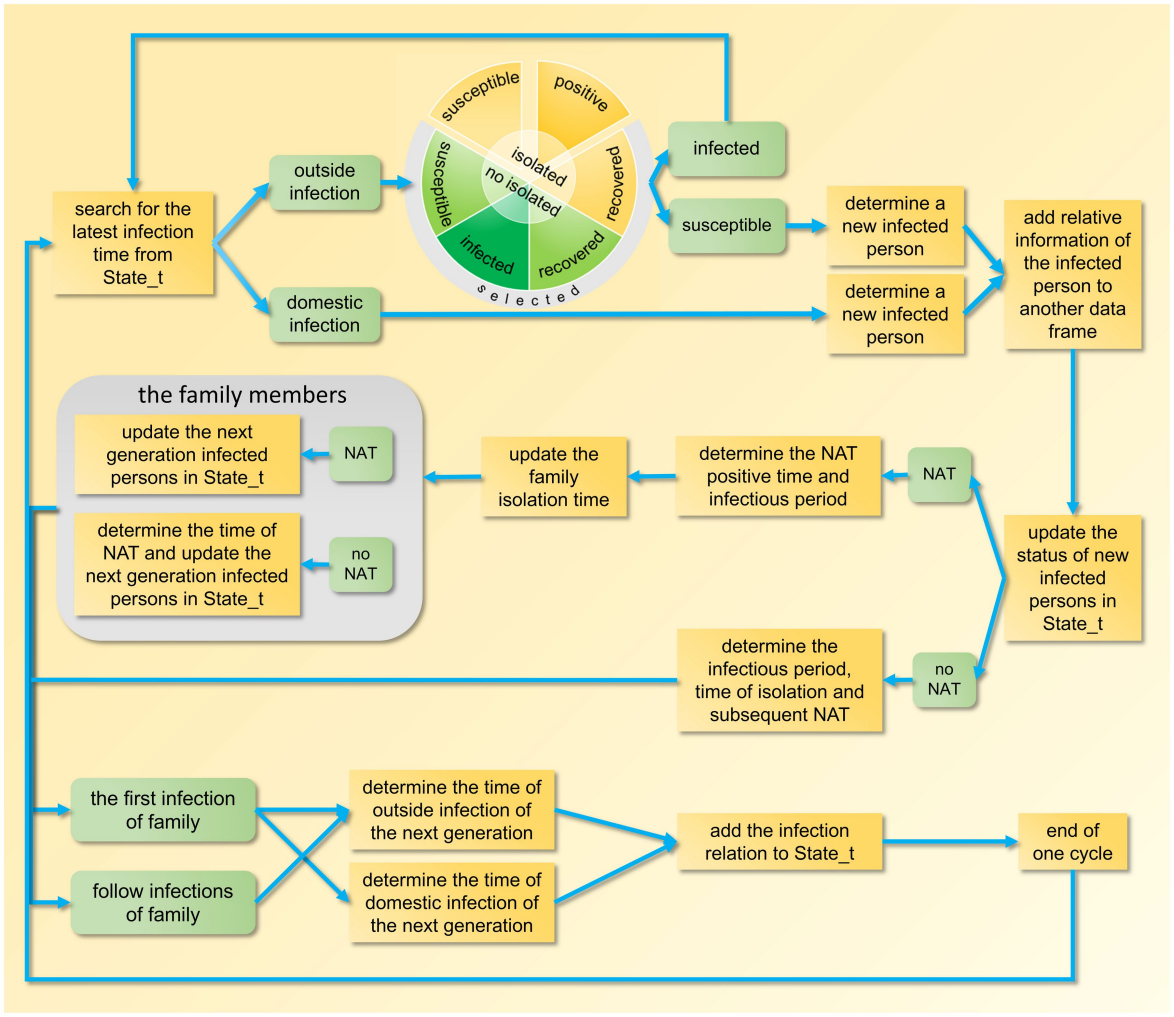
Schematic diagram of a design framework for an epidemic model. NAT, nucleic acid testing.

After the epidemic simulation concludes, the temporal distribution of the number of infected individuals, test-positive individuals, and isolated individuals is tallied (Supplementary Appendix 1, lines 545–630). The design framework for a single epidemic transmission is illustrated in Figure 2. Following the completion of 100 iterations of the main loop, the median and fluctuation range of these variables are calculated.

### Sensitivity analysis

Since this study is theoretical in nature, it is challenging to obtain survey data in the real world that aligns with the model’s preconditions, rendering it impossible for us to fit and calibrate the model using actual data. To assess the reliability and rationality of the model, sensitivity analyses were performed, focusing on seven important parameters of the model—the proportion of the population refusing testing (*p*), period of testing (*T*), extra-household adequate contact rate (*λ*), incubation period, duration from testing positive to negative, proportion of people with innate immunity (*q*), and sensitivity of testing.

We employed the Partial Rank Correlation Coefficients and Latin Hypercube Sampling (PRCC-LHS) method, an extensively used algorithm for sensitivity analysis. This method calculates correlations between a set of parameters and the model outputs after removing the linear effects of the target parameter (25). Each parameter interval was divided into *N* smaller and equal intervals, and one sample was randomly selected from each interval. These selected parameter samples were then incorporated into the model to calculate the outputs at each time point (25, 26). A series of standard coefficients representing the correlation between each parameter and the model output were computed. All analyses were performed using MATLAB R2019a software (MathWorks, Natick, MA, United States). Detailed information can be found in Supplementary Appendix 2.

## Results

### Transmission network

**Figure 3:**
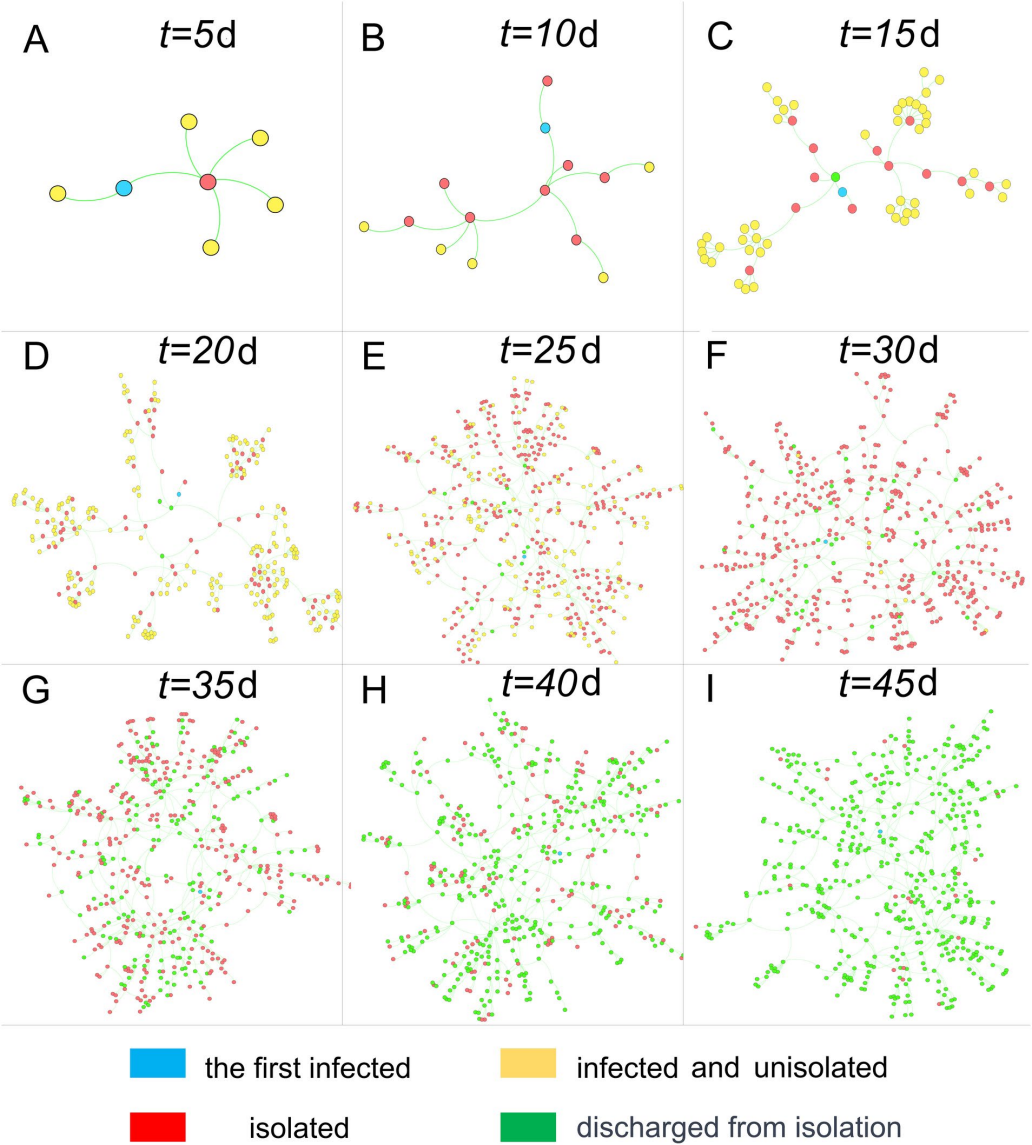
Home transmission networks at different time points. Nodes represent homes, and connections represent transmission relationships.

**Figure 4:**
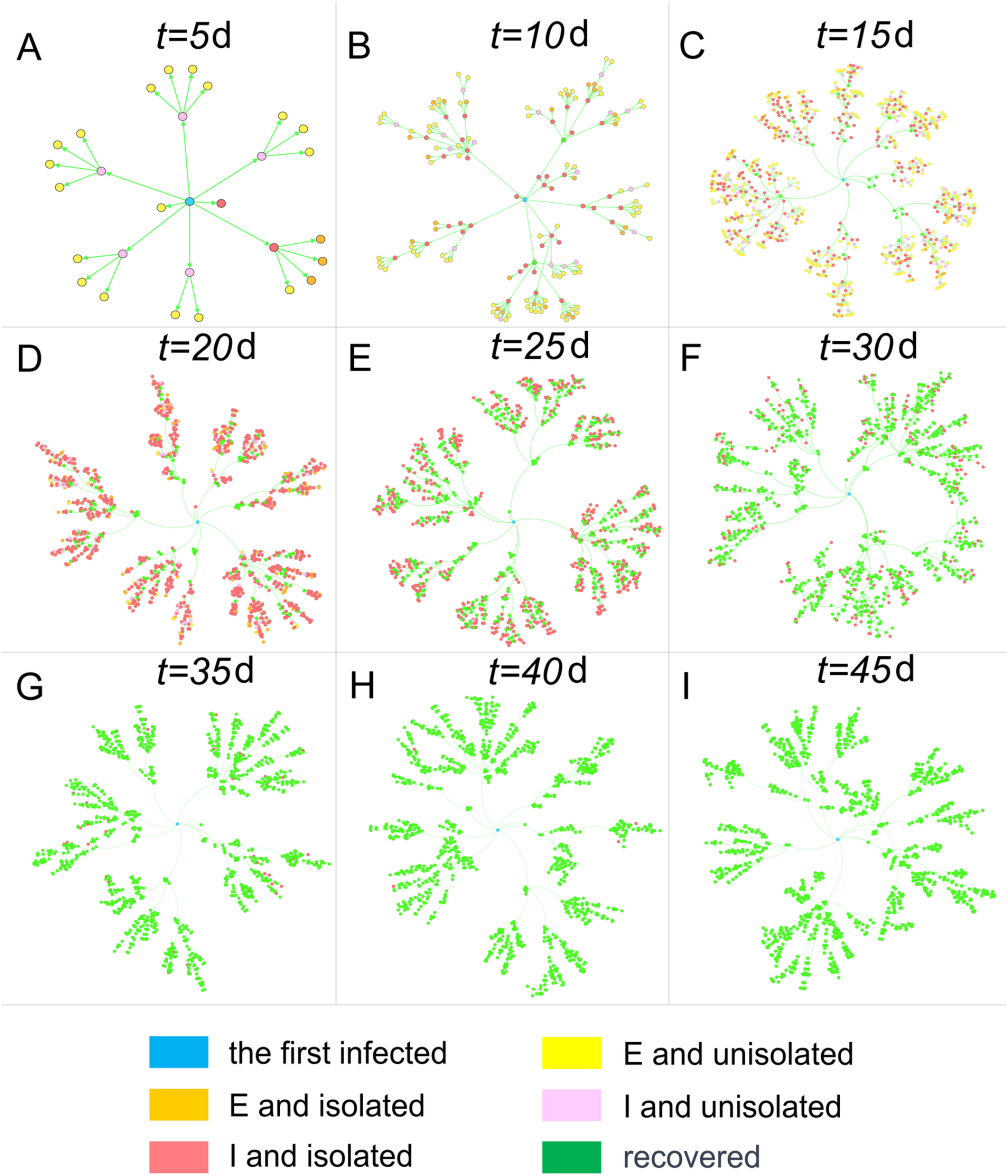
Human population transmission networks at different time points. Nodes represent individuals, and connections represent transmission relationships.

We established an infectious disease transmission network based on the epidemic’s transmission path, with homes and individuals serving as nodes and transmission relationships as links. Figures 3, 4 depict the networks for homes and individuals, respectively. Different colors are used to indicate various infection states. In Figure 3, the number of homes entering the network gradually increases over time. A home is considered infected if any of its household members are infected. Non-isolated infected homes are indicated in yellow, while homes under isolation and those that have been released from isolation are represented in red and green, respectively. As can be intuitively observed from the figure, the color of a node changes from yellow to red when the home is placed under isolation, and then turns green when all infections in the household have been released from quarantine.

In Figure 4, infected individuals are categorized into *E*, *I*, and *R*. Additionally, *E* and *I* are further distinguished based on whether the infected individuals are isolated on day *t*. The graph visually illustrates the infected individuals’ color progression from yellow (*E* and not isolated) and orange (*E* and isolated) during the incubation period to pink (*I* and not isolated) and red (*I* and isolated) during the infectious period, ultimately turning green (recovered). It is important to note that recovery is not synonymous with being released from isolation, as the latter requires a negative result from testing.

### Impact of extra-household adequate contact rate on epidemic trends

**Figure 5:**
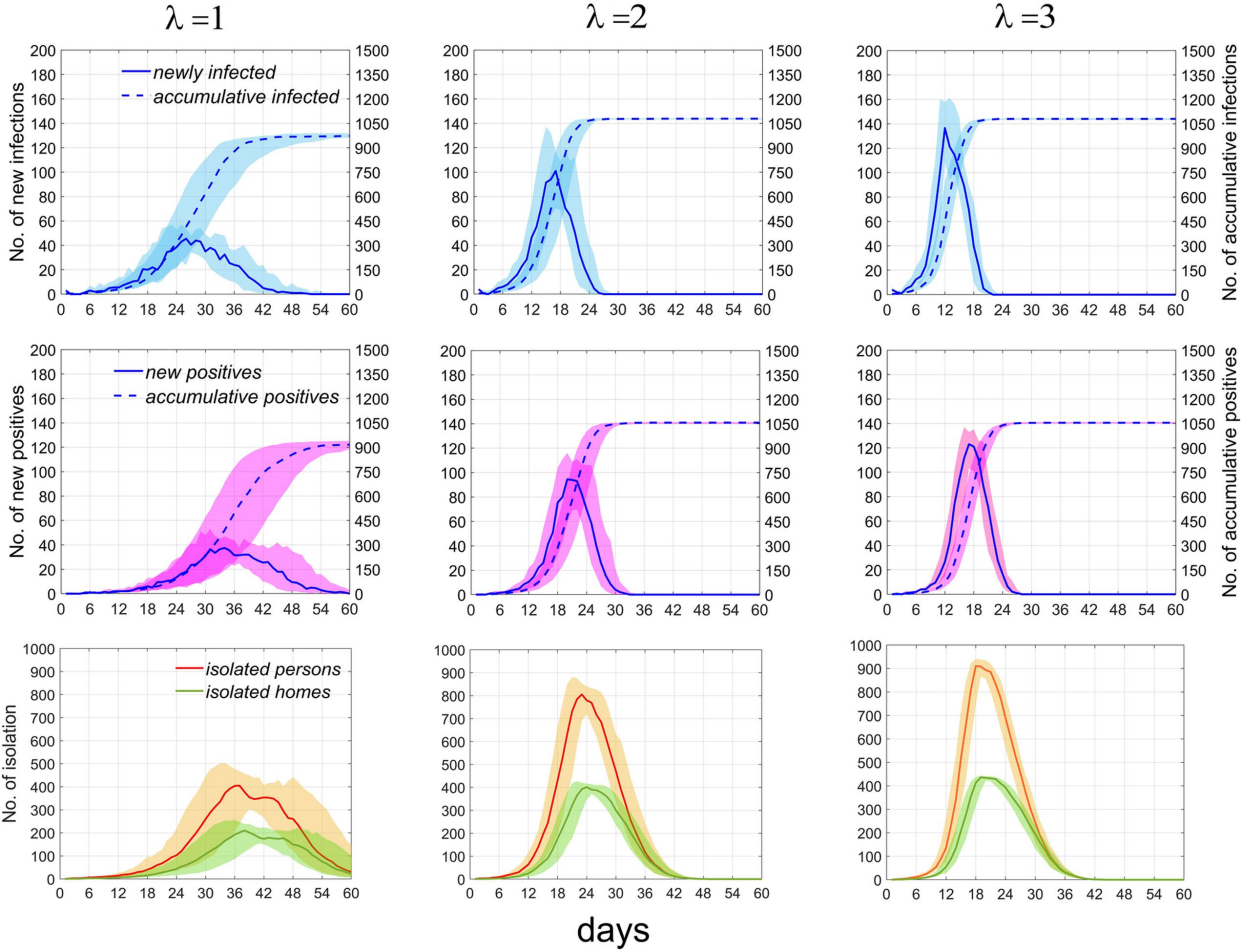
Temporal distribution of infected individuals, positive cases, and currently isolated individuals and homes under the scenario where *p* = 0.1 and *T* = 2. The first, second, and third rows represent infected individuals, positive individuals, and currently isolated individuals and households, respectively. Columns 1–3 correspond to *λ* = 1, 2, and 3, respectively. Numbers of newly infected individuals and positive individuals are indicated by the left vertical axis, while the cumulative numbers for both are denoted by the right vertical axis. Solid and dashed lines depict the median values. Areas of different colors indicate fluctuation ranges from 25 to 75%.

*λ*, as a key indicator reflecting overall social activity, can significantly influence the speed of epidemic transmission. To conduct a quantitative study on this parameter, we compared the temporal distribution characteristics of the number of infected, test-positive, and isolated individuals and households when the parameter took different values, based on 100 simulations. This is illustrated in Figure 5.

As *λ* increases from 1 to 2, the growth rate of infections significantly accelerates, the time to reach the peak shifts earlier, the peak value rises, the fluctuation range diminishes, and the cumulative number of infections increases. However, as the rate continues to increase beyond this point, the effect on promoting disease transmission weakens. It can be inferred that further increases in the rate will not result in significant changes to the time distribution of infections. Similar trends are observed in the number of positive test results, individuals currently in isolation, and isolated homes. These results suggest that at lower rates, the speed of epidemic transmission rapidly increases with increasing contact rate, but the promotional effect diminishes as the rate continues to rise. The specific values of the time distribution for these variables are presented in Table 2. When the total population size increases, Supplementary Figure S1 demonstrates that the impact of this parameter on the epidemic trend remains constant.

**Table 2:** Median and interquartile ranges for infected individuals, positive cases, and currently isolated individuals and homes.

	λ = 1		λ = 2		λ = 3	
Epidemic-related statistics	Peak number of people	Time to peak (days)	Peak number of people	Time to peak (days)	Peak number of people	Time to peak (days)
Newly infected persons	39.5 (17–47)	29	101 (70–117)	17	145 (122–175)	12
Accumulative infected persons	956.5 (934–976)	50	1078.5 (1074–1,081)	24	1,081 (1078–1,084)	19
New positive persons	37.5 (22–47)	34	94.5 (59–116)	20	125.5 (112–137)	16
Accumulative positive persons	916 (889–937)	55	1,057 (1049–1,061)	30	1049.5 (1046–1,052)	24
Isolated persons	405 (240–468)	37	805.5 (690–853)	23	925 (871–936)	18
Isolated homes	210.5 (118–238)	38	401.5 (346–416)	24	437 (425–442)	19

### The effect of test refusal rate and testing period on the epidemic trend

**Figure 6:**
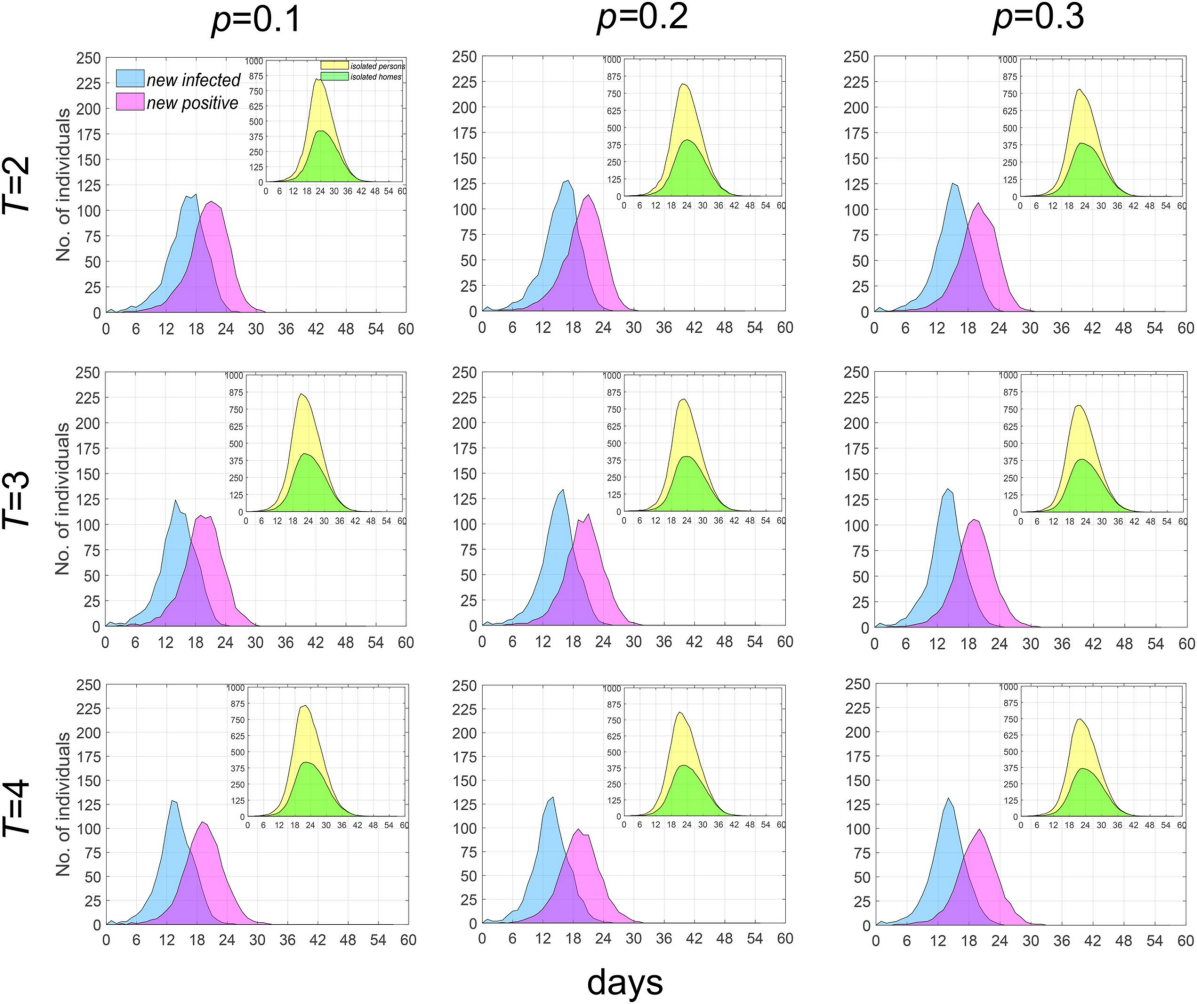
Temporal distribution of median values for newly infected individuals, positive individuals and the currently isolated individuals and homes when *λ* = 2. Infected individuals are denoted by blue, positive individuals by purple, while currently isolated individuals and homes are indicated by yellow and green, respectively.

In the early stages of an epidemic, the government promotes widespread participation in testing and strives to shorten the testing period within the constraints of testing capacity, aiming to identify infections as early and as comprehensively as possible. When the parameters *p* (representing the test refusal rate) and *T* (representing the testing period) are varied, we have analyzed the temporal distribution characteristics of new infections and new positive cases. As illustrated in Figure 6, when *T* is held constant, an increase in *p* does not significantly alter the temporal distribution of the population; the number of existing isolated households and residents decreases slightly. Conversely, when *p* is fixed, an increase in *T* leads to a slight acceleration in the growth rate of new infections, while the growth rate of new positive test results decelerates slightly; notably, the number of existing isolated households and residents remains largely unchanged. These findings indicate that the impact of increasing the coverage of nucleic acid testing and shortening the testing period on controlling epidemic spread is quite limited.

### Other epidemic characteristics

**Figure 7:**
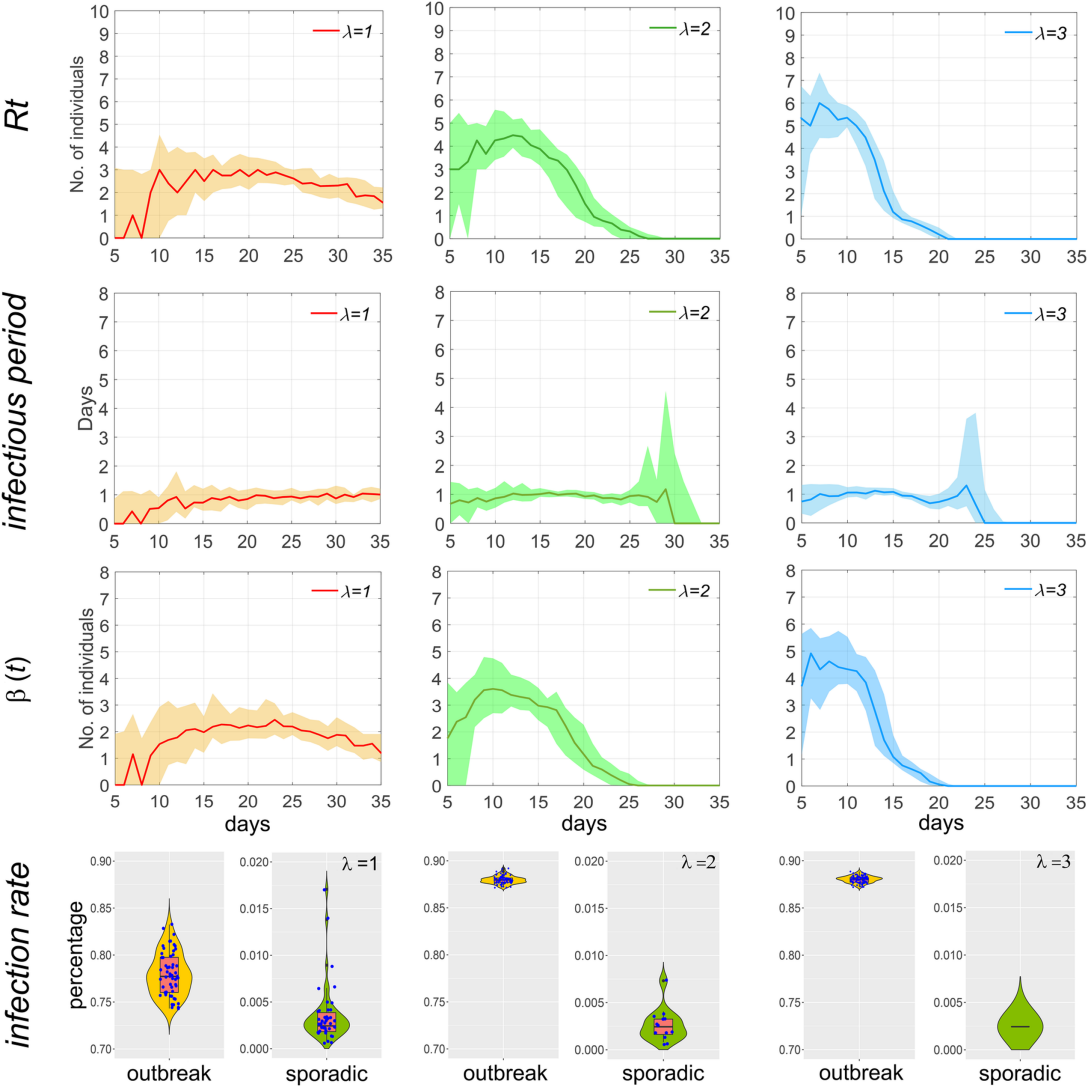
Temporal distribution of *Rt*, infectious period, *β(t)*, and the infection rate of the population during the outbreak of the epidemic when *p* = 0.1 and *T* = 2. Scatters within the violin plot represent the infection rate of the population during an epidemic.

We calculated the effective reproduction number *Rt* (i.e., the number of secondary infections transmitted by an infected individual during their infectious period at time *t*), the infectious period, and characterized the distribution of the adequate contact rate *β(t)* (the total number of secondary infections transmitted per day by an infected individual at time *t*) and the infection rate. As illustrated in Figure 7, a larger *λ* value correlates with a higher *Rt* in the early stages of the epidemic, but also with a more rapid decline; the infectious period remains relatively stable throughout the epidemic. The trend of *β(t)* is similar to that of *Rt*.

Based on the overall infection rate, the scale of the epidemic is classified into outbreaks (infection rate greater than 0.5) and sporadic cases (less than 0.5). A scatter plot is used in the fourth row of Figure 7 to visually compare the differences in incidence and infection rate between the two. Each dot represents a simulation of an epidemic. After 100 simulations, it can be observed that when *λ* = 1, the number of outbreaks is slightly higher than that of sporadic cases, and the median infection rate for sporadic cases is below 0.5‰, indicating a high likelihood of effective control of the epidemic in the initial stage through the implementation of testing and isolation. As *λ* increases, the number of outbreaks and the infection rate are significantly higher than those of sporadic cases, indicating that when *λ* exceeds 2, i.e., *R0* is greater than 3, it is difficult to fully contain the spread of the epidemic by relying solely on testing and isolation.

### Sensitivity analysis

**Figure 8:**
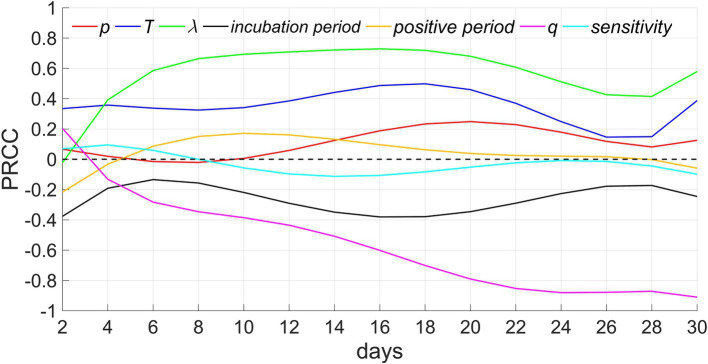
Sensitivity analysis. *p*, *T*, *λ*, and *q* denote the proportion of people who refuse testing, the testing period, the extra-household adequate contact rate, and the proportion of people with innate immunity, respectively.

In this study, we conducted sensitivity analyses on the model based on seven parameters and a continuous-time series for the total cumulative number of infections. We considered *N* = 50 samples from a uniform distribution for each parameter’s reasonable range. The PRCCs of these parameters range from-1 to 1. PRCCs close to 1 or-1 indicate that the parameter has a more positive or negative effect on the output. In contrast, a value closer to 0 indicates that the output result is less affected by the parameter (Figure 8).

Among these parameters, extra-household adequate contact rate demonstrates the greatest positive influence, the next is testing period, which is consistent with the results of Figures 5, 7. In addition, the PRCCs of test refusal rate, positive period, and sensitivity of testing fluctuate between −0.2 and 0.2, indicating that these three parameters have a relatively limited impact on the number of infectors. The impact of test refusal rate can be verified through Figure 6. In addition, because infectors are quickly isolated after testing positive, even if the positive period is extended, it will not lead to infection outside the household, hence, the influence of positive period is comparatively minor. In addition, the greater the sensitivity, the higher the accuracy of the detection, and the earlier the isolation of infectors will be implemented, thereby suppressing the spread of the epidemic. Therefore, this parameter is negatively correlated with the number of infectors, but its impact is relatively weak. The longer the incubation period, the slower the spread of the epidemic, therefore this parameter exhibits a negative correlation. The proportion of residents possessed innate immunity exhibits the strongest negative correlation, as the more immune populations there are, the more difficult it is to spread the epidemic. When herd immunity is achieved, the spread of the epidemic will cease.

## Discussion

### Innovations

This study’s strengths are mainly reflected in the following three aspects. (1) The research perspective is targeted. We have extracted a prevention and control measure of testing and isolation based on China’s practical experience in dealing with Omicron. In addition, we employed homes as a basic unit of epidemic transmission, distinguishing the locations where infections occur. (2) The research method has originality. We employed a computer model established entirely by code, with individuals as the research unit, to simulate the spread of the epidemic and the process of implementing human intervention. Through the precise design of individual behavior details, we clearly demonstrated the impact of complex prevention and control measures on the characteristics of the epidemic. (3) The model exhibits a higher level of detail. We have added numerous constraints to the model to realistically replicate the social status during the epidemic period. For instance, there are differences in infection locations between daytime and nighttime; moreover, meticulous specifications have been made regarding the timing of testing and isolation; additionally, a distinction has been drawn between the tested population and those who refuse testing. While these settings undoubtedly increase the complexity of the model design, they also improve the accuracy of analysis and prediction.

### Model parameter selection

As our model distinguishes between within-household and outside-household transmission locations, we determined the value of the extra-household adequate contact rate by referencing the *R0* values of several SARS-CoV-2 strains. Specifically, the *R0* for the original strain is approximately 2.2, while the median *R0* for the Delta and Omicron variants are around 5.08 and 10, respectively. After model tuning, we set the rate to 1–3, and the corresponding *Rt* values are illustrated in Figure 7. Additionally, recent genetic studies have revealed that the innate immune system, influenced by both environmental and genetic factors, can result in heterogeneous outcomes regarding infectivity, viral spread, and the severity and outcome of COVID-19 (27, 28). To better reflect the variability in the population’s innate immunity, we incorporated a certain proportion of non-infective individuals into our model.

### Transmission network between homes and population

In contrast to the clear tree-like features displayed by the population transmission network in Figure 4, the home transmission network in Figure 3 exhibits a mix of tree-like and disordered characteristics, owing to the close correlation between the two networks. In the population transmission network, by merging several nodes within the same home, a single node in the home transmission network is created. After traversing all homes and completing the above operations, the home transmission network is obtained. Consequently, the home transmission network retains some characteristics of the population transmission network, but it also disrupts the network structure to a certain extent.

Although many individuals have recovered (nodes turning green) in the group transmission network after the 35th day, there are still numerous homes in isolation in the home network (nodes remain red). The reasons for this are twofold: firstly, residents must undergo testing after recovery, and they can only be released from quarantine when the test result turns negative; secondly, a home can be released from quarantine only when all its members test negative, resulting in a lag time before the entire home is released from quarantine.

### The impact of extra-household adequate contact rate on the epidemic

The extra-household adequate contact rate is an indicator for measuring the speed of epidemic spread. Figure 5 and the sensitivity analysis results demonstrate that an increase in it can significantly elevate the speed and outbreak probability of the epidemic. Figure 6 reflects that the population coverage and the interval of screening have a relatively minor impact on the trend of epidemic spread. Collectively, these analyses suggest that the focus of epidemic prevention and control should be on effectively reducing the frequency of interpersonal contact, i.e., lowering the extra-household adequate contact rate, while relying solely on testing and isolation has limited effects in controlling the epidemic. The research results indicate that, in the early stages of an epidemic, measures should be promptly taken to reduce people’s contact, thereby effectively blocking transmission routes, with screening serving as a supplementary means for precise prevention and control.

### Characteristics of the time distribution of effective reproduction number, infectious period, and transmission rate

The effective reproduction number (*Rt*) is high in the early stages of an epidemic and then rapidly declines. This is because, in the initial phase, a large number of infectors come into contact with susceptible individuals, and the infectious period is prolonged. As the epidemic progresses, the number of susceptible individuals decreases, and home isolation measures shorten the infectious period, thereby increasing the resistance to epidemic spread and consequently reducing the effective reproduction number. A higher extra-household adequate contact rate results in a shorter duration of the epidemic, as a faster spread of the epidemic leads to an increase in the number of positive detections, which in turn causes a rapid increase in the number of isolated households. Once households are isolated, transmission ceases. *Rt* exhibits similar changing characteristics to *β(t)*, as *Rt* is equal to *β(t)* multiplied by the infectious period, which remains largely constant.

### Impact of assumptions on generalizability of results

In this study, we focused on exploring the association between testing, isolation, and the speed of disease transmission, and formulated seven assumptions based on the specific context of China. Although these assumptions simplify the model, they also exert a certain degree of influence on the generalizability of the results. The main impacts are as follows: Firstly, testing and isolation represent relatively stringent NPIs that are primarily suitable for controlling emerging infectious diseases with severe human harm during emergencies, such as COVID-19. Since these measures can have negative impacts on economic development and social order, many countries adopt a cautious approach toward them. Secondly, we did not consider the testing and isolation of imported cases, which may have overestimated the effectiveness of these measures in disease control. Thirdly, the model did not account for outbreaks in crowded places, such as clustered epidemics in schools, which could also affect the estimation of epidemic trends.

### The impact of other factors on the model

(1) Willingness to travel. In this model, we assume that individuals with a negative test result can move freely as long as their household is not in quarantine. However, in reality, as the epidemic spreads, people often choose to reduce outings to lower the risk of infection. Therefore, extra-household adequate contact rate would further decrease with increased social distancing. If this factor is taken into consideration, the model would underestimate the control effectiveness of testing and isolation. (2) NPIs. Following an outbreak, the government usually implements measures to limit social activities to decrease the likelihood of infection, which will also reduce extra-household adequate contact rate. Therefore, when other NPIs are combined with testing and isolation, they can have a better control effect on some more infectious strains. (3) Virus variants. The continuous mutation of the coronavirus will also produce a large number of strains with immune escape capability (29), which increases the risk of reinfection and the enhanced transmissibility. These factors will in turn change the parameter values in the model, such as extra-household adequate contact rate, the proportion of the population with innate immunity, and the hospitalization rate. These influential factors will cause changes in the output results of the model.

## Limitations

The limitations of the study are mainly reflected in the following four aspects. (1) The model’s calculations were completed using a personal computer, which is limited by computing power. Although the research conclusions remain constant when the population size increases, the relatively small population number may still weaken the persuasiveness of the conclusions to some extent. (2) The model was validated through sensitivity analysis; however, it was not calibrated utilizing actual survey data obtained from the epidemic. This is because there is a significant difference between the actual epidemic situation in China and the model design scheme. For example, when positive individuals were found, they and their close contacts were often isolated separately in government-designated locations rather than at home. (3) The design difficulty of computer models is higher than that of differential equation models. The more constraints there are, the more complex the temporal relationship of events, leading to a sharp increase in programming difficulty. Additionally, the computation speed of computer models is much slower than that of differential equation models. Personal computers may struggle when dealing with the population size of a city. (4) The study’s conclusion shows that when *R0* is less than 3, the implementation of testing and isolation has a more effective impact on epidemic prevention and control than when *R0* is greater than 3. However, due to the extremely complex system affecting the spread of infectious diseases in reality, and considering that the model is just an idealized virtual world, its research results are only applicable to the model itself. Therefore, in response to emerging infectious diseases, it is still necessary to carry out comprehensive analysis based on actual situations, and the exclusive use of this scheme needs to be particularly cautious. Furthermore, the selected measure is designed based on China’s experience and may not be applicable to some other countries.

## Conclusion

We have developed an individual-based computational model to simulate the spread of COVID-19 in a small town, and quantitatively evaluated the efficacy of population-wide testing, isolation and treatment for infected individuals, as well as home quarantine for close contacts. The model results indicate that this strategy can significantly decrease the infection rate and slow down the transmission speed when the disease spreads at a lower rate. However, even with increased coverage of testing and shortened testing intervals, the epidemic cannot be fully contained, and a substantial number of households would still be subject to quarantine. Therefore, the emphasis of epidemic prevention and control should be on effectively minimizing person-to-person contact and boosting the population’s immune level. To this end, in addition to screening and isolation, it is also imperative to restrict the number of individuals in public places, promote remote working or teaching, enhance personal protective measures and environmental disinfection, and undertake comprehensive strategies such as large-scale population immunization.

## Data Availability

The datasets presented in this study can be found in online repositories. The names of the repository/repositories and accession number(s) can be found in the article/Supplementary material.

## Supplementary material

Supplementary Figure S1 The temporal distribution of the number of infected individuals when the population size varies (DOI: 10.17632/wbwfnvps6j.2).

Supplementary Appendix 1 The program of evaluating the impact of large-scale nucleic acid testing and home quarantine on COVID-19 prevention and control: a dynamic modeling approach (DOI:10.17632/pz4n372r9g.2).

Supplementary Appendix 2 The sensitivity analysis of evaluating the impact of large-scale nucleic acid testing and home quarantine on COVID-19 prevention and control: a dynamic modeling approach (DOI:10.17632/vz56g9jtfh.2).
