# Supplementary material for: Evaluating the impact of large-scale nucleic acid testing and home quarantine on a novel emerging infectious disease prevention and control: a dynamic modeling approach
Source: Front Public Health. 2025 May 19;13:1447738. doi: 10.3389/fpubh.2025.1447738 (PMC12127344; doi:10.3389/fpubh.2025.1447738)
Supplement: Supplementary Appendix 1 — The program of evaluating the impact of large-scale nucleic acid testing and home quarantine on COVID-19 prevention and control: a dynamic modeling approach (DOI:10.17632/pz4n372r9g.2). [file Table_1.docx]

%%%

%%%

%%%

%%%

% Basic parameter settings

clear all

nf=500; % Number of households

n_serial=[0.2,0.33,0.28,0.19]*nf;

np=[1,2,3,4]*n_serial'; % Total number of people

mu_inc=3.1; sd_inc=2.6; % incubation period

mu_hos=6.35; sd_hos=2.5; % Hospitalization time

mu_pos=8.4; sd_pos=4.8; % Duration of nucleic acid positivity

p_antibody=0.1; % Proportion of people with immunity

p_antitest=0.3; % Proportion of people who do not test for nucleic acid

int_test=4; % Nucleic acid testing cycle (days)

lambda=2; % External infection index

cycle=100; % Number of cycles

T=100; % Iteration deadline

sensitivity=0.87; % Sensitivity of PCR nucleic acid detection

Home=[]; % Store personal numbers by home

New_E=[]; % Store the number of new infected individuals

New_P=[]; % Store the number of new positive cases

Isof=[]; % Number of storage isolated households

Isop=[]; % Number of storage isolation personnel

TT=[];

Rt=[]; % Time variation of storing basic regeneration numbers

Inf_P=[]; % Storage contagion period

Lambda=[]; % Storage external infection index

unepi=[]; % Storing the number of infected individuals before a pandemic occurs

% Establish a family distribution matrix Home, where the k-th row represents the index of the family members of the k-th family

n_serial_cum=cumsum(n_serial);

n_house_cum=cumsum([n_serial(1),n_serial(2)*2,n_serial(3)*3]);

% The second column after represents the family member number

Home(1:n_serial_cum(1),1)=[1:n_house_cum(1)]';

for i=1:n_serial(2)

Home(n_serial_cum(1)+i,1:2)=[n_house_cum(1)+(i-1)*2+1:n_house_cum(1)+(i-1)*2+2];

end

for i=1:n_serial(3)

Home(n_serial_cum(2)+i,1:3)=[n_house_cum(2)+(i-1)*3+1:n_house_cum(2)+(i-1)*3+3];

end

for i=1:n_serial(4)

Home(n_serial_cum(3)+i,1:4)=[n_house_cum(3)+(i-1)*4+1:n_house_cum(3)+(i-1)*4+4];

end

%no_state stores population numbers with immunity

no_antibody=randperm(np);

no_antibody=no_antibody(1:round(p_antibody*np));

no_state=zeros(1,np);

no_state(no_antibody)=1;

%no_test storages of population numbers without nucleic acid testing

no_antitest=randperm(np);

no_antitest=no_antitest(1:round(p_antitest*np));

no_test=zeros(1,np);

no_test(no_antitest)=1;

% Start systemic circulation

for i=1:cycle

i

% Initialize State, store personal infection status according to home, corresponding to Home

[s1,s2]=size(Home);

State=zeros(s1,s2);

State(find(Home>0))=1;

for j=1:length(no_antibody)

State(find(Home==no_antibody(j)))=6;

end

% Set the number 0 infected person as index 0, have no immunity, and undergo nucleic acid testing

index1=randperm(np);

index0=index1(1);

no_tem=no_state+no_test;

while no_tem(index0)>0

index0=randperm(np);

index0=index0(1);

end

[rH,cH]=find(Home==index0);

D=[];

rD=0;

State_t=[];

% Store the infection time inside and outside the household, line 1 (1 within the household, 2 outside the household); Line 2: Family of the infected person;

% Line 3 is the source of infection; Line 4 Infected person; Line 5 infection time

% Initialize State_t with index0

State_t(1,1)=2;

State_t(2,1)=rH;

State_t(3,1)=0;

State_t(4,1)=index0;

State_t(5,1)=-3;

[rS,cS]=size(State_t);

t_hp=zeros(nf,1);

isolations=zeros(1,np);

while cS>0

% Find the vector that was first infected in State_t

ftm=find(State_t(5,:)==min(State_t(5,:)));

state0=State_t(:,ftm);

State_t(:,ftm)=[];

t=state0(5);

%Incubation period

inc_per=normrnd(mu_inc, sd_inc);

while inc_per<1 | inc_per>5

inc_per=normrnd(mu_inc, sd_inc);

end

% Determine the duration of nucleic acid positivity, which refers to the duration from the first positive test to the first negative test

inf_per=normrnd(mu_pos, sd_pos);

while inf_per<3

inf_per=normrnd(mu_pos, sd_pos);

end

% D: 1. Source of infection index, 2. Infected person, 3. Infected family, 4. Whether the family is infected (1 yes, 2 no), 5. Infection time,

% 6. Start of infection time, 7. Test positive time, 8 time to enter the shelter, 9 time to turn negative, and 10 time to release isolation

run0=0;

if state0(1)==1

rD=rD+1;

D(rD,1)=state0(3); D(rD,2)=state0(4); D(rD,3)=state0(2); D(rD,4)=state0(1);

D(rD,5)=state0(5); D(rD,6)=D(rD,5)+inc_per; D(rD,9)=D(rD,6)+inf_per; D(rD,10)=D(rD,9)+rand*int_test;

index0=D(rD,2);

[rH,cH]=find(Home==index0);

else

if rD>0

isolations=zeros(np,1);

% Find the index of all accessible individuals outside of the family at time t, and find the next generation of contacts from these individuals

% Family members of infectious agents

[rs0,cs0]=find(Home==state0(3));

isolations(Home(rs0,find(Home(rs0,:)>0)))=1;

% Find the family that has been quarantined at time t

iso_fams=find(t_hp>0);

for j=1:length(iso_fams)

reva=Home(iso_fams(j),find(Home(iso_fams(j),:)>0));

isolations(reva)=1;

for k=1:length(reva)

if t>D(find(D(:,2)==reva(k)),10)

% If the iso_ The reva (k) member of Fams (j) family turned negative at time t

isolations(reva(k))=0;

end

end

end

nexts=find(isolations==0);

ran_next=randperm(length(nexts));

index0=nexts(ran_next(1));

[rH,cH]=find(Home==index0);

if State(rH,cH)==1

rD=rD+1;

D(rD,1)=state0(3); D(rD,2)=index0; D(rD,3)=rH; D(rD,4)=state0(1);

D(rD,5)=state0(5); D(rD,6)=D(rD,5)+inc_per; D(rD,9)=D(rD,6)+inf_per; D(rD,10)=D(rD,9)+rand*int_test;

else

run0=1;

end

else

rD=rD+1;

D(rD,1)=state0(3); D(rD,2)=state0(4); D(rD,3)=state0(2); D(rD,4)=state0(1);

D(rD,5)=state0(5); D(rD,6)=0; D(rD,9)=D(rD,6)+inf_per; D(rD,10)=D(rD,9)+rand*int_test;

end

end

% Further improve the information after D (rD, 7)

if run0==0

State(rH,cH)=3;

% Nucleic acid testing time

% Time of first positive nucleic acid test

thp0=0;

if no_test(index0)==0

% Determine the time of nucleic acid positivity t_ test1

temp_inf=D(rD,6)-floor(D(rD,6));

r_test=randperm(int_test);

d_test=r_test(1);

ran_t=rand*(17/24-8/24)+8/24;

if d_test==1

if temp_inf<=ran_t

t_test1=floor(D(rD,6))+ran_t;

else

t_test1=floor(D(rD,6))+int_test+ran_t;

end

else

t_test1=floor(D(rD,6))+d_test-1+ran_t;

end

% Number of nucleic acid tests during the infection period

p_test=binornd(1,sensitivity);

times_test=1;

while p_test==0

p_test=binornd(1,sensitivity);

times_test=times_test+1;

end

if times_test>1

t_test1=floor(t_test1)+(times_test-1)*int_test+ran_t;

end

if t_test1<=D(rD,9)

D(rD,7)=t_test1;

t_h=rand+1;

D(rD,8)=D(rD,7)+t_h;

else

D(rD,7)=0;

D(rD,8)=0;

t_test1=T+1;

end

% Determine the infection period t_ Min and number of infected individuals

% Update the earliest positive time for nucleic acid testing in the family t_ hp

if t_hp(rH)~=0

if floor(t_test1)<floor(t_hp(rH))

t_min=floor(t_test1)+19/24;

t_hp(rH)=t_test1;

thp0=1;

else

if D(rD,9)<t_test1

if D(rD,9)<t_hp(rH)

t_min=D(rD,9);

elseif D(rD,6)<t_hp(rH) & t_hp(rH)<=D(rD,9)

t_min=floor(t_hp(rH))+19/24;

elseif t_hp(rH)<=D(rD,6)

t_min=0;

end

else

if D(rD,6)<t_hp(rH) & t_hp(rH)<=D(rD,9)

t_min=floor(t_hp(rH))+19/24;

elseif t_hp(rH)<=D(rD,6)

t_min=0;

end

end

end

else

if D(rD,9)<t_test1

t_min= D(rD,9);

elseif floor(t_test1)+19/24<D(rD,9)

t_min=floor(t_test1)+19/24;

t_hp(rH)=t_test1;

thp0=2;

elseif D(rD,9)<=floor(t_test1)+19/24

t_min=D(rD,9);

t_hp(rH)=t_test1;

thp0=2;

end

end

%Find unknown nucleic acid carriers who have previously been infected with the same family

inf_pro=D(find(D([1:rD-1],3)==rH),2);

inf1=[];

inf0=[];

for j=1:length(inf_pro)

if no_test(inf_pro(j))==1;

inf1=[inf1,inf_pro(j)];

else

inf0=[inf0,inf_pro(j)];

end

end

% Determine the relationship between t_ hp (rH) and the time of negative conversion in individuals without nucleic acid testing

if length(inf1)>0

for j=1:length(inf1)

rinf=find(D(:,2)==inf1(j));

d_inf=D(rinf,9);

if (thp0==1 | thp0==2) & t_hp(rH)<d_inf

% People who do not undergo nucleic acid testing begin to undergo nucleic acid testing every two days,

% starting from the second day of home quarantine

p_test0=binornd(1,sensitivity);

times_test0=1;

while p_test0==0

p_test0=binornd(1,sensitivity);

times_test0=times_test0+1;

end

t_test0=ceil(t_hp(rH))+(times_test0-1)*int_test+rand*(17/24-8/24)+8/24;

if t_test0<d_inf

D(rinf,7)=t_test0;

t_fc=t_test0+rand+1;

D(rinf,8)=t_fc;

% Delete the second generation infected individuals in the family after entering the shelter

f1=find(State_t(1,:)==1 & State_t(3,:)==inf1(j) & State_t(5,:)>D(rinf,8));

if length(f1)>0

State_t(:,f1)=[];

end

f1=find(State_t(1,:)==2 & State_t(3,:)==inf1(j) & State_t(5,:)>floor(t_hp(rH))+19/24);

if length(f1)>0

State_t(:,f1)=[];

end

end

end

end

end

% Determine the relationship between t_ hp (rH) and nucleic acid testers

if length(inf0)>0

for j=1:length(inf0)

rinf=find(D(:,2)==inf0(j));

d_inf=D(rinf,9);

if (thp0==1 | thp0==2) & t_hp(rH)<d_inf

f_rem=find(State_t(1,:)==2 & State_t(3,:)==inf0(j) & State_t(5,:)>t_hp(rH));

if length(f_rem)>0

State_t(:,f_rem)=[];

end

end

end

end

else

if t_hp(rH)>0

if D(rD,9)<floor(t_hp(rH))+19/24

D(rD,7)=0; D(rD,8)=0;

t_min=D(rD,9);

else

p_test0=binornd(1,sensitivity);

times_test0=1;

while p_test0==0

p_test0=binornd(1,sensitivity);

times_test0=times_test0+1;

end

ran_t0=rand*(17/24-8/24)+8/24;

if D(rD,6)<t_hp(rH)

t_test0=ceil(t_hp(rH))+(times_test0-1)*int_test+ran_t0;

t_min=floor(t_hp(rH))+19/24;

else

t_test0=ceil(D(rD,6))+(times_test0-1)*int_test+ran_t0;

t_min=0;

end

%1. Infectious source index, 2. Infected person, 3. Infected family, 4. Whether the family is infected (1 yes, 2 no),

% 5. Infection time, 6. Start of infection, 7. Test positive time, 8. Enter the shelter, 9. Turn negative time,

% 10. Release of isolation time

if t_test0<D(rD,9)

D(rD,7)=t_test0;

t_fc=t_test0+rand+1;

D(rD,8)=t_fc;

else

D(rD,7)=0;

D(rD,8)=0;

end

end

else

D(rD,7)=0;

D(rD,8)=0;

t_min=D(rD,9);

end

end

TT=[TT;D(rD,2),floor(t_hp(rH))+19/24,t_min];

% Number and duration of transmission of infected individuals outside the household during the infection period (daily time span: 7/24-19/24)

if D(rD,6)<t_min

t_serials=[];

temp_inf=D(rD,6)-floor(D(rD,6));

% The t th_ Infected person and time before min

if temp_inf<7/24

inf_days=floor(t_min)-floor(D(rD,6));

t_start=floor(D(rD,6))+7/24;

for k=1: inf_days

no_inf=poissrnd(lambda*(1-sum(isolations)/np));

t_serial=[];

if no_inf>0

t_serial=sort(rand(1,no_inf)*(19/24-7/24))+t_start;

end

t_start=t_start+1;

t_serials=[t_serials,t_serial];

end

elseif temp_inf>=7/24 & temp_inf<19/24

inf_days=floor(t_min)-floor(D(rD,6));

t_start=ceil(D(rD,6)+0.0000001)+7/24;

for k=1: inf_days

if k==1

no_inf=poissrnd(lambda*(1-sum(isolations)/np)*(19/24-temp_inf)/(19/24-7/24));

t_serial=[];

if no_inf>0

t_serial=sort(rand(1,no_inf)*(19/24-temp_inf))+D(rD,6);

end

t_serials=[t_serials,t_serial];

else

no_inf=poissrnd(lambda*(1-sum(isolations)/np));

t_serial=[];

if no_inf>0

t_serial=sort(rand(1,no_inf)*(19/24-7/24))+t_start;

end

t_start=t_start+1;

t_serials=[t_serials,t_serial];

end

end

elseif temp_inf>=19/24

inf_days=floor(t_min)-ceil(D(rD,6));

t_start=ceil(D(rD,6)+0.0000001)+7/24;

for k=1:inf_days

no_inf=poissrnd(lambda*(1-sum(isolations)/np));

t_serial=[];

if no_inf>0

t_serial=sort(rand(1,no_inf)*(19/24-7/24))+t_start;

end

t_start=t_start+1;

t_serials=[t_serials,t_serial];

end

end

% Supplementing the number of infected individuals and infection time at t_min

temp_min=t_min-floor(t_min);

if temp_min<7/24

inf_day=0;

elseif temp_min>=7/24 & temp_min<19/24

inf_day=temp_min-7/24;

elseif temp_min>=19/24

inf_day=19/24-7/24;

end

no_inf=poissrnd(lambda*(1-sum(isolations)/np)*inf_day/(19/24-7/24));

if no_inf>0

last_inf=sort(rand(1,no_inf)*inf_day)+floor(t_min)+7/24;

t_serials=[t_serials,last_inf];

end

if length(t_serials)>0

State_h=[];

% Store the time of infection outside the home, line 1 (1 within the home, 2 outside the home); Line 2: Family of the infected person;

% Line 3 is the source of infection; Line 4 Infected person; Line 5 infection time

State_h(5,:)=t_serials;

State_h(1,:)=2;

State_h(2,:)=0;

State_h(3,:)=D(rD,2);

State_h(4,:)=0;

State_t=[State_t,State_h];

end

end

% Number of infections transmitted within households during the infection period

if thp0==2 & sum(State(rH,:)==3)==1

% Determine the end time of the infection period inf_ over

if D(rD,7)==0

inf_over=D(rD,9);

else

inf_over=D(rD,8);

end

% Number of second-generation infected individuals in households

nu_s=round(sum(State(rH,:)==1));

% Individuals infected within the family and their duration of infection

if nu_s>0

% Determine the time of second-generation infection

temp_over=inf_over-floor(inf_over);

if temp_inf<19/24

if temp_inf<7/24

start1=D(rD,6);

over1=floor(D(rD,6))+7/24;

inf1=over1-start1;

else

start1=[];

over1=[];

inf1=0;

end

if temp_over<7/24

days2=floor(inf_over)-floor(D(rD,6))-1;

else

days2=floor(inf_over)-floor(D(rD,6));

end

start2=[]; over2=[]; inf2=0; intervals2=[];

if days2>0

for m=1:days2

start2=[start2,floor(D(rD,6))+19/24+m-1];

over2=[over2,ceil(D(rD,6)+0.0000001)+7/24+m-1];

end

inf2=days2*12/24;

intervals2=ones(1,days2)*12/24;

end

if temp_over<7/24

start3=floor(inf_over)-1+19/24;

over3=inf_over;

inf3=over3-start3;

elseif temp_over>=7/24 & temp_over<19/24

start3=[];

over3=[];

inf3=0;

elseif temp_over>=19/24

start3=floor(inf_over)+19/24;

over3=inf_over;

inf3=over3-start3;

end

elseif temp_inf>=19/24

start1=D(rD,6);

over1=ceil(D(rD,6)+0.0000001)+7/24;

inf1=over1-start1;

if temp_over<7/24

days2=floor(inf_over)-floor(D(rD,6))-2;

else

days2=floor(inf_over)-floor(D(rD,6))-1;

end

start2=[]; over2=[]; inf2=0; intervals2=[];

if days2>0

for m=1:days2

start2=[start2,floor(D(rD,6))+19/24+m-1];

over2=[over2,ceil(D(rD,6))+7/24+m-1];

end

inf2=days2*12/24;

intervals2=ones(1,days2)*12/24;

end

if temp_over<7/24

start3=floor(inf_over)-1+19/24;

over3=inf_over;

inf3=over3-start3;

elseif temp_over>=7/24 & temp_over<19/24

start3=[];

over3=[];

inf3=0;

elseif temp_over>=19/24

start3=floor(inf_over)+19/24;

over3=inf_over;

inf3=over3-start3;

end

end

infs=[]; to_int=[]; starts=[]; overs=[];

if inf1>0

infs=[infs,inf1];

to_int=[to_int,inf1];

starts=[starts,start1];

overs=[overs,over1];

elseif inf2>0

infs=[infs,inf2];

to_int=[to_int,intervals2];

starts=[starts,start2];

overs=[overs,over2];

elseif inf3>0

infs=[infs,inf3];

to_int=[to_int,inf3];

starts=[starts,start3];

overs=[overs,over3];

end

inf_times=sort(rand(1,nu_s)*sum(infs));

cum_int=cumsum(to_int);

times=[];

for m=1:nu_s

n=1;

while inf_times(m)>cum_int(n)

n=n+1;

end

if n==1

times=[times,starts(n)+inf_times(m)];

else

times=[times,starts(n)+inf_times(m)-cum_int(n-1)];

end

end

% Determine the index of second-generation infected individuals

susceptibles=Home(rH,find(State(rH,:)==1));

rank_s=randperm(length(susceptibles));

susceptibles=susceptibles(rank_s);

if length(susceptibles)>0

%Establish State_h

State_h=[];

State_h(4,:)=susceptibles;

State_h(1,:)=1;

State_h(2,:)=rH;

State_h(3,:)=D(rD,2);

State_h(5,:)=times;

State_t=[State_t,State_h];

end

end

end

State_t(:,find(State_t(4,:)==D(rD,2)))=[];

end

[rS,cS]=size(State_t);

end

if rD>=0.5*np

% Number of new infected and positive cases

tmax=ceil(max(D(:,10)));

for k=1:tmax

New_E(i,k)=sum(k-1<D(:,5) & D(:,5)<=k);

New_P(i,k)=sum(k-1<D(:,7) & D(:,7)<=k);

end

% Family isolation situation

t_ho=zeros(nf,1);

for j=1:nf

t_over=D(find(D(:,3)==j),10);

if length(t_over)>0

t_ho(j)=max(t_over);

end

end

for j=1:tmax

m=0;

for k=1:nf

if t_hp(k)>0 & ceil(t_hp(k))<=j & j<=floor(t_ho(k))

m=m+1;

end

end

Isof(i,j)=m;

end

% Personnel isolation situation

iso_fams=find(t_hp>0);

for nt=1:tmax

m=0;

for j=1:length(iso_fams)

reva=find(D(:,3)==iso_fams(j));

for k=1:length(reva)

if floor(t_hp(iso_fams(j)))+19/24<nt & nt<D(reva(k),10)

m=m+1;

end

end

end

Isop(i,nt)=m;

End

% Distribution of Rt and Lambda

PR=[];

for j=1:np

Di=D(find(D(:,1)==j),:);

in_inf=sum(Di(:,4)==1);

out_inf=sum(Di(:,4)==2);

if sum(find(D(:,2)==j))>0

t_inf=D(find(D(:,2)==j),6);

[rH,cH]=find(Home==j);

if t_inf<floor(t_hp(rH))+19/24 & t_hp(rH)>0

per_inf=min(floor(t_hp(rH))+19/24,D(find(D(:,2)==j),9))-t_inf;

elseif floor(t_hp(rH))+19/24<t_inf & t_hp(rH)>0

per_inf=0;

elseif t_hp(rH)==0

per_inf=D(find(D(:,2)==j),9)-t_inf;

end

PR=[PR;[j,t_inf,per_inf, out_inf,in_inf]];

end

end

PR0=PR(find(PR(:,3)>0),:);

t=PR0(:,2)+0.5*PR0(:,3);

for j=1:ceil(tmax)

PR00=PR0(find(j-1<=t & t<j),:);

[rR,cR]=size(PR00);

if rR>0

Rt(i,j)=mean(PR00(:,4)+PR00(:,5));

Lambda(i,j)=mean((PR00(:,4)+PR00(:,5))./PR00(:,3));

else

Rt(i,j)=0;

Lambda(i,j)=0;

end

end

%infectious period

t=PR(:,2)+0.5*PR(:,3);

for j=1:ceil(tmax)

PR00=PR(find(j-1<=t & t<j),:);

[rR,cR]=size(PR00);

if rR>0

Inf_P(i,j)=mean(PR00(:,3));

else

Inf_P(i,j)=0;

end

end

else

unepi=[unepi,length(D(:,2))];

end

end
